# Phytoremediation of Oil-Contaminated Soil by *Tagetes erecta* L. Combined with Biochar and Microbial Agent

**DOI:** 10.3390/plants14020243

**Published:** 2025-01-16

**Authors:** Xin Fang, Pufan Zheng, Haomin Wang, Kefan Wang, Cong Shi, Fuchen Shi

**Affiliations:** 1 College of Life Sciences, Nankai University, Tianjin 300071, China; 1120210521@mail.nankai.edu.cn (X.F.); whmnku@mail.nankai.edu.cn (H.W.); kfwang3333@163.com (K.W.); 2 Institute of Agricultural Products Preservation and Processing Technology (National Engineering and Technology Research Center for Preservation of Agricultural Products), Tianjin Academy of Agricultural Sciences, Key Laboratory of Storage and Preservation of Agricultural Products, Ministry of Agriculture and Rural Affairs, Tianjin Key Laboratory of Postharvest Physiology and Storage and Preservation of Agricultural Products, Tianjin 300384, China; zhengpufan@foxmail.com; 3 School of Environmental Science and Engineering, Tiangong University, Tianjin 300387, China

**Keywords:** bioremediation, petroleum-degrading bacteria, biochar, phytoremediation, oil-contaminated soil

## Abstract

Crude oil pollution of soil is an important issue that has serious effects on both the environment and human health. Phytoremediation is a promising approach to cleaning up oil-contaminated soil. In order to facilitate phytoremediation effects for oil-contaminated soil, this study set up a pot experiment to explore the co-application potentiality of *Tagetes erecta* L. with two other methods: microbial agent and biochar. Results showed that the greatest total petroleum hydrocarbon (TPH) biodegradation (76.60%) occurred in the soil treated with *T. erecta*, a microbial agent, and biochar; the highest biomass and root activity also occurred in this treatment.GC-MS analysis showed that petroleum hydrocarbon components in the range from C_10_ to C_40_ all reduced in different treatments, and intermediate-chain alkanes were preferred by our bioremediation methods. Compared with the treatments with biochar, the chlorophyll fluorescence parameter NPQ_Lss and plant antioxidant enzyme activities significantly decreased in the treatments applied with the microbial agent, while soil enzyme activities, especially oxidoreductase activities, significantly increased. Although the correlation between biochar and most plant growth and soil enzyme activity indicators was not significant in this study, the interaction effect analysis found a synergistic effect between microbial agents and biochar. Overall, this study suggests the co-addition of microbial agents and biochar as an excellent method to improve the phytoremediation effects of oil-contaminated soil and enhances our understanding of the inner mechanism.

## 1. Introduction

Oil pollution is a kind of complex and serious organic pollution and attracts attention worldwide. Oil spills during production and transport can contaminate soil and water sources and damage soil structure [1]. The toxicity of crude oil also has adverse effects on the survival of microorganisms, animals, and plants in the soil [2]. Tang et al. found that oil pollution inhibited the growth of bacteria in the soil, as well as the germination rate and root growth of plants [3]. Hentati et al. observed that the survival rate of earthworms also dramatically decreased with increasing oil concentration [4]. In addition, various toxic substances in oil can also enter the food chain system through groundwater and crops, ultimately endangering human health [5]. Therefore, the remediation of oil-contaminated soil needs to be paid great attention and studied urgently.

Currently, the remediation techniques for soil oil contamination become more diverse, including physical remediation, chemical remediation, and bioremediation. Due to the high cost and serious secondary pollution of physical and chemical remediation, bioremediation technology has become an increasingly hot topic for researchers since it was developed in the 1940s [6,7]. And phytoremediation is one of the most common bioremediation methods, which utilizes plants, especially their root systems, to remove pollutants from the soil and restore contaminated soil. Phytoremediation mainly involves four different mechanisms according to the process: phytostabilization, phytodegradation, phytovolatilization, and rhizodegradation [8,9]. Phytoremediation has been widely practiced in soil pollution remediation, including heavy metals and organic pollutants. The plants applied in pollution remediation usually have the characteristics of large roots and high transpiration rates, which are also present in many ornamental plants such as *Tagetes erecta* [10]. Various species of plants in the Asteraceae family have been reported to perform well in the remediation of heavy metals in soil [11]. In terms of organic pollution, Kambhu et al. also found three plants, including *T. erecta*, could increase the removal efficiency of styrene in soil [12].

Phytoremediation has the advantages of being economical and eco-friendly, but in practical applications, it is often used in association with other restoration methods for higher restoration efficiency and wider environmental adaptability. One of the methods often combined with phytoremediation is microbial remediation. Under oil pollution, plant growth, development, and photosynthesis are negatively affected [13]. But in plant–microorganism combined remediation, microorganisms can degrade organic pollutants and reduce stress, thereby promoting plant growth [14]. Meanwhile, plants can also support microbial growth through rhizosphere effects [15,16]. In addition, another commonly used method in combination with phytoremediation is soil amendment, such as biochar. Biochar, as an adsorbent material produced by biomass pyrolysis, has been widely used in the remediation of various types of pollution, including petroleum pollution [17]. Biochar is a soil amendment that can store carbon relatively steadily. And in the remediation of soil oil pollution, biochar also serves as a microbial carrier and an immobilizing agent of pollutants, assisting in plant remediation [18,19].

Enzymes also play an important role in the remediation of soil contamination and can act as a qualified ecological indicator to monitor the recovery of soil quality [20]. Enzymes in soil are derived mainly from soil microorganisms, plant root exudates, and the decomposition of animal and plant residues [21]. These enzymes perform a wide variety of functions in the soil, participating in processes such as organic matter decomposition, nutrient transformation, and cycling, which are crucial for maintaining soil fertility and ecosystem stability [22,23]. Many organic pollutants, due to their complex structure and poor water solubility, usually require enzyme catalysis before they can be ultimately absorbed and degraded by microorganisms [24,25]. The enzymes that function in pollution remediation mainly include hydrolases and oxidoreductases [26]. Hydrolases are mainly involved in the mineralization process of organic matter, hydrolyzing large molecules into easily absorbed and utilized small molecules, such as phosphatase and urease [27,28]. Oxidoreductases, such as dehydrogenases and catalases, mainly catalyze the redox reactions of hydrogen transfer and electron transfer and have been applied to degrade various natural or manmade pollutants [29,30]. This study aims to address three issues in the combined remediation of soil petroleum pollution using microbial agents and biochar-assisted plants: how microorganisms and biochar promote the degradation of petroleum by plants; how effectively different remediation methods degrade different components of petroleum; and how plant and soil enzymes respond under different remediation methods.

## 2. Results

### 2.1. Total Petroleum Hydrocarbons (TPH) Degradation Efficiency Under Different Remediation Methods

**Figure 1:**
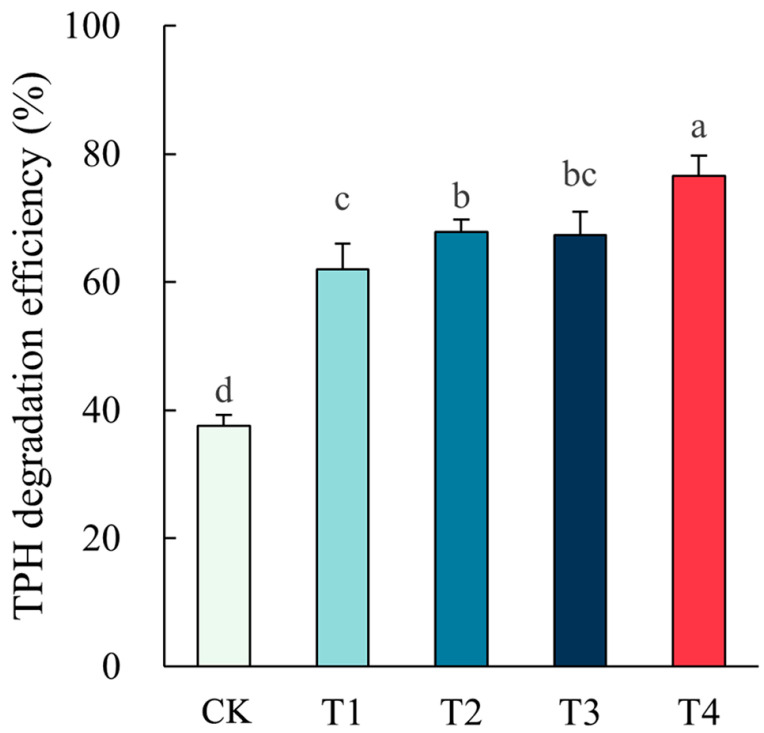
TPH degradation efficiency and effects of different remediation methods. Different letters indicate significant differences in TPH degradation efficiency between different methods.

**Figure 2:**
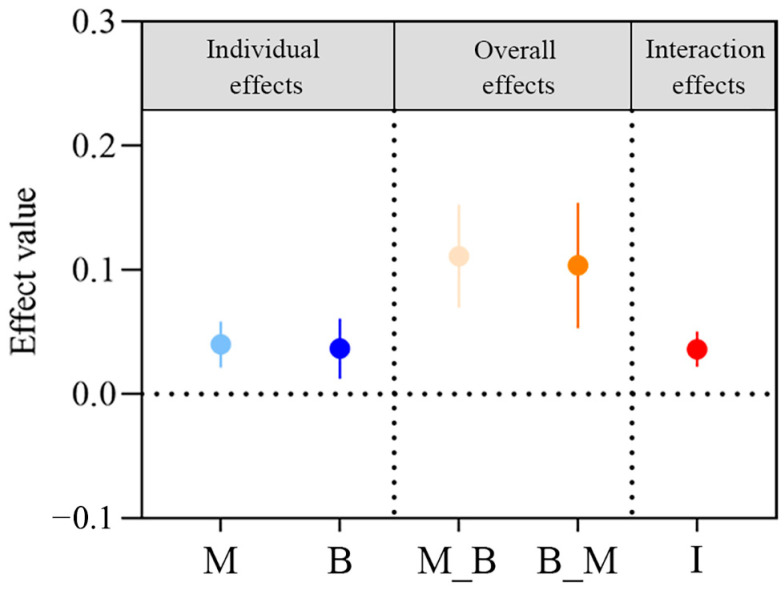
Individual, overall, and interaction effects of microbial agent (M) and biochar (B) on phytoremediation. M: the individual effect of microbial agent; B: the individual effects of biochar; M_B: the overall effects of microbial agent across the presence and absence of biochar; B_M: the overall effects of biochar across the presence and absence of microbial agent; I: the interaction effects of microbial agent and biochar.

Single phytoremediation and combinations with microbial agents or biochar all showed significant remediation effects of oil-contaminated soils, with TPH degradation efficiency significantly higher than those of unremediated soils (Figure 1). The highest TPH degradation efficiency of 76.60% occurred in the combined application of plant, microbial agent, and biochar, which was elevated significantly by 1.04 folds versus the CK treatment (*p* < 0.05). The enhancement effect of microbial agents and biochar on phytoremediation is shown in Figure 2. The enhancement effect of microbial agents was higher than that of biochar in both individual effects and overall effects. The positive value of interaction effects demonstrated that the combined treatment of microbial agent and biochar had a higher enhancement effect on phytoremediation than the sum of their individual effects, indicating a synergistic effect between the two methods.

### 2.2. Soil Petroleum n-Alkane Components Under Different Remediation Methods

**Figure 3:**
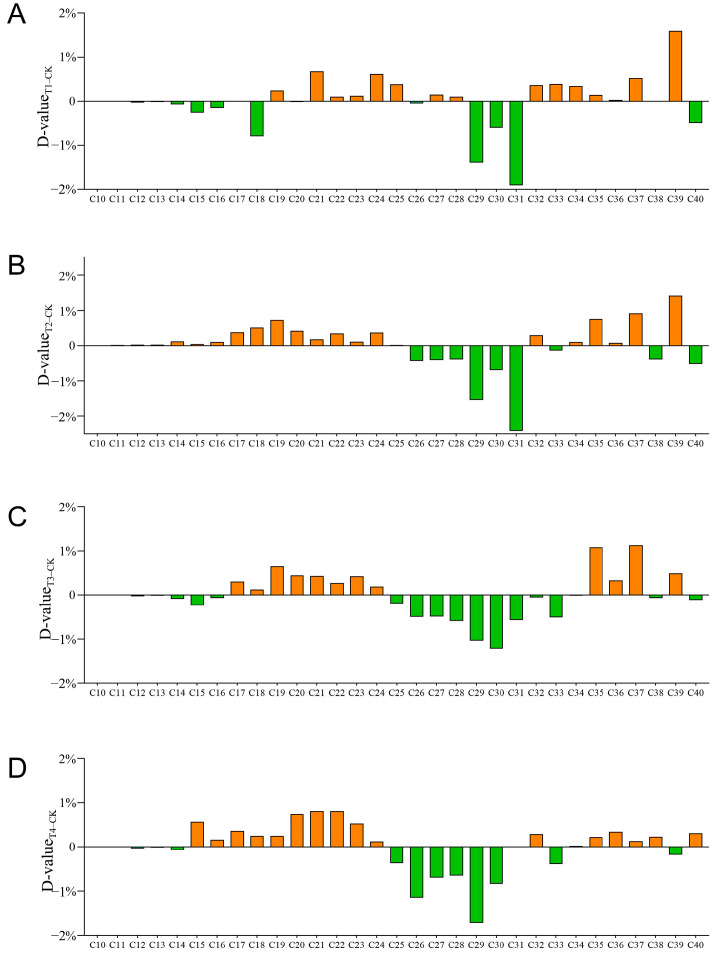
Changes in relative abundance of n-alkanes between different treatments: (**A**) CK and T1; (**B**) CK and T2; (**C**) CK and T3; (**D**) CK and T4. The vertical coordinate indicates the relative changes, which were expressed as D-values between the values of relative abundance in the two treatments, and color represents increase or decrease (orange or green). The horizontal coordinate indicates the types of n-alkanes during C_10_–C_40_.

The GC-MS chromatograms of petroleum hydrocarbon components in contaminated soils of five treatments are shown in Figure S1. And Figure 3 shows the changes in the relative abundance of detected n-alkanes from C_10_ (decane) to C_40_ (tetracontane). In the remediated soils (T1–T4), the content of components in the range of C_10_ to C_12_ components was almost undetectable. In addition, the trends of changes in other components were similar between the repaired treatments and control, except for differences in the carbon number range of the components with increased or decreased relative content. Taking the T4 treatment with the highest TPH degradation efficiency as an example (Figure 3D), the relative abundance of extremely short-chain alkanes (C_10_–C_14_) decreased compared to control, the relative abundance of short-chain alkanes (C_15_–C_24_) increased, and the relative abundance of intermediate-chain alkanes (C_25_–C_30_) decreased. The relative abundance changes in long-chain alkanes (C_31_–C_40_) were not obvious, mostly higher than the control and a few lower. This result suggested that after remediation, the content of the short- and long-chain saturated alkanes decreased relatively little, while the intermediate-chain saturated alkanes decreased the most. Compared with the T4 treatment, more short-chain alkanes (C_12_–C_18_) were reduced in relative abundance in the T1 treatment, and more intermediate-chain alkanes (C_25_–C_33_) were reduced in the T3 treatment. In addition, other treatments (T1, T2, and T3) had higher elevated relative abundances of long-chain alkanes, such as C_37_ and C_39_, than the T4 treatment.

### 2.3. Plant Growth Under Different Remediation Methods

**Figure 4:**
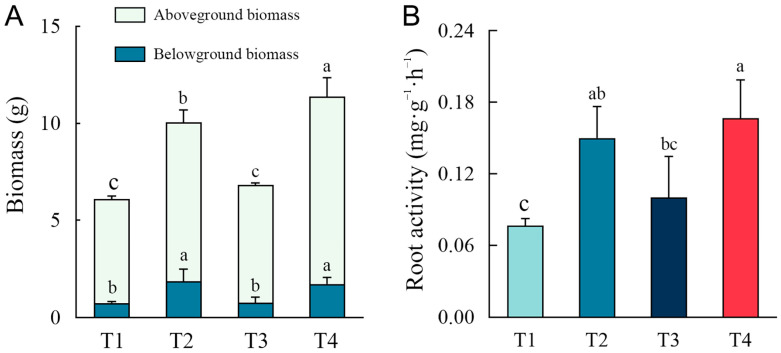
Biomass (**A**) and root activity (**B**) of plants with different remediation methods. Different letters indicate significant differences between different methods.

The growth status of the plants at day 90 is shown in Figure S2, and the biomass and root activity of plants after remediation are shown in Figure 4. Compared to the single phytoremediation treatment (T1), the addition of biochar (T3) had no significant effect on the increase in belowground and aboveground biomass of plants, whereas the treatments with the addition of a microbial agent (T2 and T4) had a significant increase in plant biomass. The aboveground biomass and belowground biomass of T2 were significantly increased by 53.18% and 146.35%, respectively, compared to T1 (*p* < 0.05), and the aboveground biomass and belowground biomass of T4 were significantly increased by 80.29% and 129.65%, respectively, compared to T1 (*p* < 0.05). It can be seen that the microbial agent addition gave a more significant enhancement of belowground biomass than aboveground biomass. In addition, treatments containing microbial agents (T2 and T4) also significantly improved plant root activity, while the effect of biochar on plant root activity was lower, similar to the changes in biomass.

### 2.4. Soil Enzyme Activity Under Different Remediation Methods

**Figure 5:**
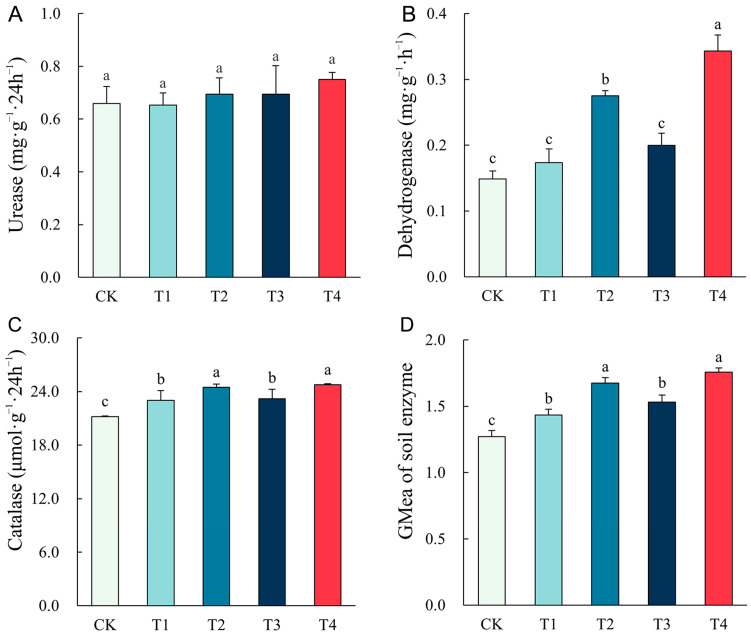
Effect of different remediation methods on the activities of soil enzymes: (**A**) urease; (**B**) dehydrogenase; (**C**) catalase; (**D**) GMea of soil enzyme. Different letters indicate significant differences between different methods (*p* < 0.05).

The soil enzyme activities under different remediation methods are shown in Figure 5. Urease activity was not significantly affected by remediation methods, and no significant difference was observed. But the activity of two oxidoreductases, dehydrogenase and catalase, showed significant differences between different treatments. The control soil (CK) without remediation possessed the lowest enzyme activities, while the treatment with the combination of plants, microbial agents, and biochar (T4) had the highest dehydrogenase and catalase activities, which increased by 130.78% and 16.71%, respectively, compared to T1 (*p* < 0.05). The treatment with the co-application of plants and microbial agents (T2) also enhanced the dehydrogenase and catalase activities significantly (*p* < 0.05). And GMea of soil enzyme showed a similar trend.

### 2.5. Plant Antioxidant Enzyme Activity Under Different Remediation Methods

**Figure 6:**
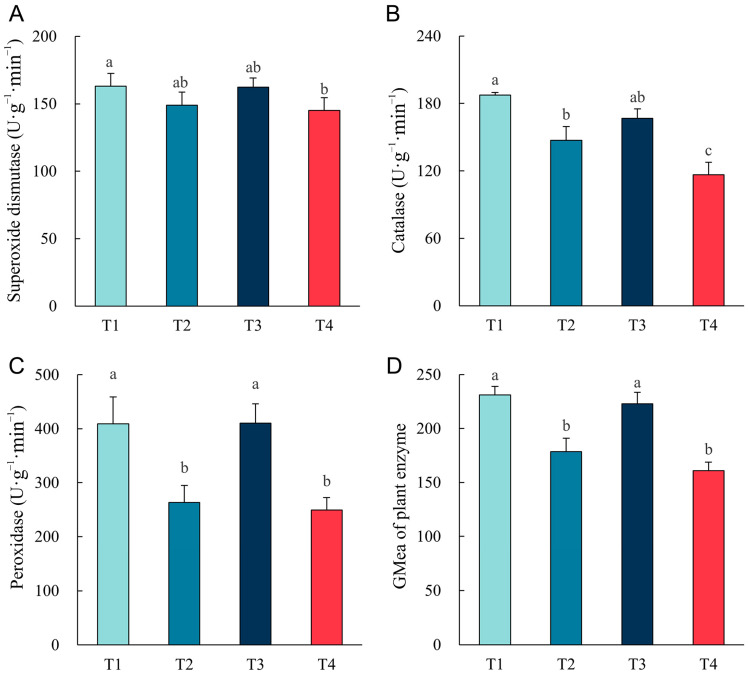
Effect of different remediation methods on the activities of plant antioxidant enzymes: (**A**) superoxide dismutase (SOD); (**B**) catalase (CAT); (**C**) peroxidase (POD); (**D**) GMea of plant enzyme. Different letters indicate significant differences between different methods (*p* < 0.05).

The plant antioxidant enzyme activities under different remediation methods are shown in Figure 6. Changes in plant antioxidant enzyme activities were opposite to soil oxidoreductase activities. The activities of three antioxidant enzymes, SOD, CAT, and POD, were higher in the treatments of single phytoremediation (T1) and plant biochar co-remediation (T3). In contrast, antioxidant enzyme activities were significantly lower in T2 treatment and T4 treatment, featuring high TPH degradation rates and good plant growth (*p* < 0.05). GMea of soil enzyme was also significantly higher in the T1 and T3 treatments than in the T2 and T4 treatments (*p* < 0.05).

### 2.6. Plant Chlorophyll Fluorescence Under Different Remediation Methods

**Figure 7:**
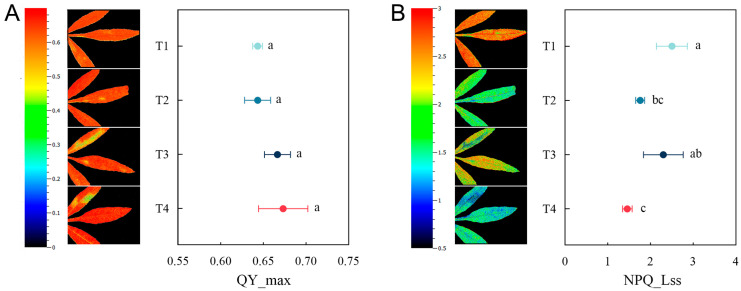
Changes in chlorophyll fluorescence parameters among different remediation treatments: (**A**) QY_max (maximum light quantum yield); (**B**) NPQ_Lss (steady-state non-photochemical fluorescence quenching). Different letters indicate significant differences between different treatments (*p* < 0.05). The color scales alongside the fluorescence images correspond to the QY_max and NPQ_Lss values, and the colors from blue to red indicate increasing values.

The values and imaging of chlorophyll fluorescence parameters QY_max and NPQ_Lss of *T. erecta* under different treatments are shown in Figure 7. QY_max (maximum light quantum yield) can reflect the potential maximum photosynthetic capacity of plants. There was no significant difference in QY_max values and imaging among the four treatments, all within a healthy range. NPQ_Lss (steady-state non-photochemical fluorescence quenching) represents the excess energy that plants dissipate in non-photochemical forms such as thermal energy under stress and is a self-protection mechanism of plants. Among the four treatments, T1 had the highest NPQ_Lss value, followed by T3. NPQ_Lss was significantly lower in treatments T2 and T4 with the addition of a microbial agent than in the single phytoremediation treatment (T1). In addition, chlorophyll fluorescence imaging of T3 and T4 showed that NPQ_Lss was higher at the leaf base than at the leaf tip.

### 2.7. Correlation Analysis Between Remediation Methods and Physiological Indicators

**Figure 8:**
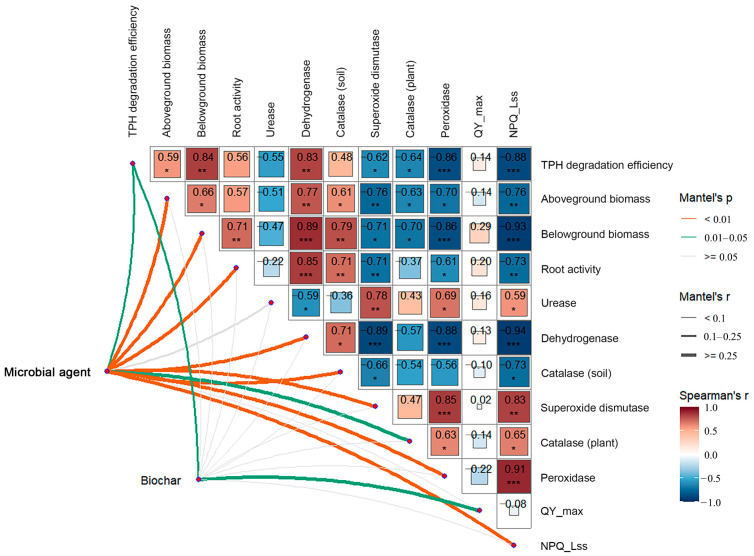
Correlation analysis between remediation methods and biochemical indicators. The color gradient corresponded to Spearman’s correlation coefficients (Spearman’s r) between different indicators, and significant correlations were indicated by asterisks (* *p* < 0.05, ** *p* < 0.01, *** *p* < 0.001). Microbial agents and biochar were related to each index by Mantel tests. Edge width denotes Mantel’s r value, and edge color denotes the statistical significance (Mantel’s *p*). The orange and green lines represented significant correlations at the levels of 0.01 and 0.05, respectively.

From the correlation analysis in Figure 8, it can be seen that the soil TPH degradation efficiency was significantly positively correlated with plant aboveground biomass (*p* < 0.05), belowground biomass (*p* < 0.01), and soil dehydrogenase activity (*p* < 0.01), and significantly negatively correlated with plant antioxidant enzyme activity (*p* < 0.05 or *p* < 0.001), and NPQ_Lss (*p* < 0.001). In addition, a significantly positive correlation was found between soil oxidoreductases (dehydrogenase and catalase) and plant growth parameters (aboveground biomass, belowground biomass, root activity), whereas plant antioxidant enzyme (SOD, CAT, and POD) activities were significantly negatively correlated with plant growth parameters. The chlorophyll fluorescence parameter QY_max was not significantly correlated with other physiological indicators, but NPQ_Lss was significantly negatively correlated with plant growth parameters and positively correlated with plant antioxidant enzyme activities. The addition of a microbial agent had a significant impact on all indicators except urease, whereas biochar was only significantly associated with TPH degradation efficiency and QY_max.

## 3. Discussion

In this study, the addition of a microbial agent and biochar both improved the remediation effect of phytoremediation on soil oil pollution. Especially with the combined use of microbial agents, biochar, and plants, the TPH degradation efficiency reached the highest level (76.6%). Petroleum n-alkanes from C_10_ to C_40_ were all degraded to varying degrees in this study, with intermediate-chain alkanes degraded the most (C_25_–C_30_). Xu et al. also observed the highest degradation in C_24_–C_29_ alkanes by soil-indigenous bacteria in oil-contaminated soil [31]. Due to the high toxicity of short-chain alkanes and the low solubility and bioavailability of long-chain alkanes, bioremediation usually prefers intermediate-chain alkanes [32]. Notably, the increase in the relative abundance of long-chain alkanes under the T4 treatment was lower than the other treatments. This may suggest that the combined application of microbial agents, biochar, and plants has a better remediation effect on long-chain alkanes and is more likely to avoid the accumulation of long-chain alkanes in the remediated soil.

Oil pollution is a form of adversity stress for plants. *T. erecta* was found as one of the hydrocarbon-tolerant plants, but its individual remediation in high-concentration oil-contaminated soil was ineffective, and its growth was also affected [33]. In addition, based on the comparisons and analyses in our previous studies, we found that *T. erecta* was more likely to show better remediation effects with combined remediation methods and had the potential to enrich *Bacillus* in microbial agents in the rhizosphere zone [34]. In this study, microbial agents promoted plant biomass and root activity to a greater extent than biochar. Meanwhile, in the microbial agent addition treatments with higher biomass and root activity recovery, both plant antioxidant enzyme activities and chlorophyll fluorescence index NPQ_Lss decreased. Plant antioxidant enzymes are an important mechanism by which plants remove active oxygen and defend against adversities [35]. And chlorophyll fluorescence tools can detect tolerance and acclimation responses of plants under stress [36]. QY_max is an effective index for evaluating the growth status and degree of stress of plants [37]. NPQ_Lss is a photoprotection strategy in which plants dissipate excess energy that cannot be utilized by the photosynthetic system through non-photochemical pathways such as thermal energy [38]. Correlation analysis found a significant correlation between plant antioxidant enzyme activities and NPQ_Lss. Both of these indicators reflect the degree of plant stress by oil pollution, and both are means for plants to decrease oxidative stress. The enhancement of antioxidant enzymes can catalyze the conversion of ROS, and the increase in NPQ_Lss can manage the over-excitation of PSII, thereby preventing greater oxidative stress [39].

Plant-microbe interactions are widely thought of as an important process influencing the efficiency of the phytoremediation of oil-contaminated soil. By investigating the changes in soil and plant enzyme activities, the changes in plant growth parameters, the changes in chlorophyll fluorescence parameters, and their correlations, this study revealed that one of the mechanisms by which microbial agents improved phytoremediation lay in the enhancement of the activities of soil oxidoreductase, thus lowering the concentration of petroleum in the soil, decreasing the degree of plant stress by pollution, and promoting the growth of plants. This was verified by the elevation of soil oxidoreductase (dehydrogenase, catalase) activities and plant growth parameters, as well as the reduction in the plant antioxidant enzyme activities and NPQ_Lss in the treatments with the addition of the microbial agent. Other studies have also shown that the combined use of microorganisms and plants could induce the synthesis of some oxidative enzymes [32]. In the study of Xun et al. [40], the activities of both plant antioxidant enzymes and soil enzymes increased in the phytoremediation of oil-contaminated soil with a combination of plant growth-promoting bacteria (PGPR) and arbuscular mycorrhizal fungi (AMF). And they considered that PGPR and AMF could make the plants more tolerant to petroleum stress. In our study, microbial agents increased the activities of soil oxidoreductases but decreased the activities of plant antioxidant enzymes. This may stem from the different pathways through which plant growth-promoting bacteria and petroleum-degrading bacteria function. Petroleum-degrading bacteria are more likely to degrade petroleum in soil through the petroleum-degrading ability of the bacteria and the promotion of soil enzyme activities, which could lead to a reduction in petroleum stress on plants. In addition, we also found that oxidoreductases were more sensitive to oil pollution stress than hydrolases. Wyszkowska and Wyszkowski also found in the experiment that urease was the enzyme with the smallest activity change under oil pollution [41]. Compared to hydrolases, oxidoreductases are more likely to be the “core enzyme” that plays a critical role in the degradation of pollutants. This has been corroborated in remediation research for other pollutant types [40]. Moreover, as one of the oxidoreductases, dehydrogenase is often considered an indicator of overall microbial activity as well [42].

Different researchers have different opinions about the role of biochar in oil pollution remediation. Some literature suggested that the adsorption function of biochar could reduce soil oil concentration, decrease nutrient leaching, provide shelter for microorganisms, and thus enhance TPH removal efficiency [43,44]. However, it has also been found that the adsorption of organic pollutants by biochar led to a decrease in the bioavailability of the pollutants, which could have a negative effect on the degradation of TPH [45,46]. In this study, the addition of biochar promoted the degradation of soil oil contamination by *T. erecta*. But unlike microbial agents, the correlations between biochar and most plant growth and soil enzyme activity indicators were not significant. The explanation for this result may be that without the addition of oil-degrading bacteria, the biochar mainly relies on its own large surface area, porous structure, and interaction between the surface functional groups and pollutants to absorb petroleum hydrocarbons and reduce the TPH concentration in soil [47]. The interaction effect, however, indicated a synergistic effect between the microbial agent and biochar. Dike et al. comprehensively evaluated the effectiveness of biochar in combination with various other bioremediation methods and concluded that biochar was more effective in combined remediation than in individual remediation, especially when combined with bioaugmentation [48]. In the co-application of microbial agents with biochar, microorganisms can be adsorbed in the pores of biochar. In this case, biochar provides shelter and nutrients for soil microorganisms while microorganisms also change the adsorption environment of biochar, so that improving the biodegradation environment of soil petroleum together [49]. Therefore, the combined addition of microbial agents and biochar may be an excellent method to enhance phytoremediation, and we recommend the co-application of *T. erecta*, biochar, and microbial agents as a possible remediation method for oil-contaminated soil.

## 4. Materials and Methods

### 4.1. Experiment Treatments and Design

The culture soil used in the experiment was provided by the Beijing Jiahui Landscaping and Flower Company (Beijing, China) and sterilized by autoclaving before the experiment. Crude oil was provided by Dagang Oilfield (Tianjin, China). A total of 1.5 kg of culture soil and 5.5 g of crude oil were added into pots and mixed thoroughly so that the petroleum concentration in the soil was close to the oil-contaminated soil samples from the Dagang oil field. There were five treatments in this experiment: oil-contaminated soil (CK); oil-contaminated soil planted with *T. erecta* (T1); oil-contaminated soil planted with *T. erecta* and added with a microbial agent (5% dry weight of soil) (T2); oil-contaminated soil planted with *T. erecta* and added with biochar (4% dry weight of soil) (T3); oil-contaminated soil planted with *T. erecta* and added with a microbial agent and biochar (the same additive amounts as above) (T4). The microbial agent was produced by Yangzhou Hairuike Co., Ltd. (Yangzhou, China) and was a lyophilized formulation whose main components were oil-degrading bacteria of *Bacillus* and *Micrococcus*, together with enzymes and nutrients. Biochar was produced from corn stover and provided by Henan Lize Environmental Protection Technology Co., Ltd. (Zhengzhou, China). *T. erecta* seedlings of healthy and similar growth were transplanted into pots after germination. Each treatment had 3 replicates. Each pot had 3 seedlings and was watered every day to keep the soil moisture at ~15%. The experiment was carried out in a greenhouse with natural temperature and sunlight for 90 days from June to September 2022. Samples were taken on day 90.

### 4.2. Detection of Soil Total Petroleum Hydrocarbon

Soil samples were air-dried and passed through a 2 mm sieve after being taken from the pots on day 90. Soil samples were stored at 4 °C for subsequent analysis. A total of 30 g soil samples were dissolved in 100 mL dichloromethane and extracted in an ultrasonic water bath (200 W) for 15 min. The suspension was then centrifuged at 4000 rpm for 10 min, and the supernatant was completely evaporated at 54 °C. Total petroleum hydrocarbons (TPH) content was calculated by the gravimetric method. And the degradation efficiency of TPH was calculated using the following equation [46]:

TPH Degradation efficiency (%) = (TPH_day0_ − TPH_day90_)/TPH_day0_ × 100.

Individual, overall, and interaction effects of microbial agents and biochar on phytoremediation were calculated through TPH degradation efficiency (abbreviated as E in the following equation) as follows:

Individual effect of microbial agent = ln E_T2_ − ln E_T1_.

Individual effect of biochar = ln E_T3_ − ln E_T1_.

Overall effect of microbial agent = ln (E_T2_ + E_T4_) − ln (E_T1_ + E_T3_).

Overall effect of biochar = ln (E_T3_ + E_T4_) − ln (E_T1_ + E_T2_).

Interaction effect of microbial agent and biochar = ln E_T4_ − ln E_T2_ − ln E_T3_ + ln E_T1_.

Individual effects measure an agent’s influence in isolation, while overall effects measure its influence across levels of another agent. And for interaction effects, the positive or negative values indicate that the interaction tends to increase or decrease the degradation efficiency of TPH [50].

### 4.3. Analysis of Petroleum Hydrocarbon Components

The TPH extracts were purified by silica gel and neutral alumina column chromatography. The n-alkanes were eluted with 20 mL n-hexane and concentrated to ~1 mL at 40 °C. Gas chromatography-mass spectrometry (GC-MS) was used to identify and measure the concentration of petroleum hydrocarbon components using an Agilent 7890A GC connected to a 5975C mass spectrometer (Agilent Technologies, Santa Clara, CA, USA). The GC-MS analysis conditions were as follows:

Column: HP–5MS (30.0 m × 0.25 mm × 0.25 μm).

GC conditions: split-less injection was used with an injection volume of 1 μL; the inlet temperature was 280 °C; the heating program was 60 °C for 2 min, 30 °C/min to 200 °C, and 5 °C/min to 320 °C for 10 min; carrier gas was helium with a flow rate of 1 mL/min.

MS conditions: the mass spectrum was recorded in electron impact (EI) mode and full scan mode (*m*/*z* range 14–650); the ion source temperature was 230 °C; the quadrupole temperature was 150 °C.

According to Atai et al. [51], relative quantitative analysis for each n-alkane component was carried out by integrating the peak at a particular *m*/*z*. External multi-level calibrations were performed using n-alkane (C_10_–C_40_) standard solutions (Anpel Laboratory Technologies, Shanghai, China) with concentrations of 1 mg/mL.

Relative changes in n-alkanes between different treatments were expressed as differences (D-value) between the values of relative abundance (R) as follows:

D-value_Tn–CK_ = R_Tn_ − R_CK_.

### 4.4. Analysis of Plant Growth Performance

On day 90 after planting, the sixth fully developed leaf from the top of *T. erecta* was chosen to measure chlorophyll fluorescence parameters by Portable Handy FluorCam (Ecotech Ecological Technology Co., Ltd., Pleasanton, CA, USA). The measurements were performed after 20 min of dark adaptation wrapped with tin foil paper. And two chlorophyll fluorescence parameters were recorded in this study: maximum light quantum yield (QY_max) and steady-state non-photochemical fluorescence quenching (NPQ_Lss).

Fresh roots were dug for the measurement of root activity on day 90. Root activity was assayed using the 2,3,5-triphenyltetrazolium chloride (TTC) method following the procedure of Ruf and Brunner [52]. After the measure of biochemical parameters, plants were harvested and separated as shoots and roots; aboveground and belowground biomass were recorded after being dried for three days at 80 °C.

### 4.5. Analysis of Enzyme Activity

Fresh leaf samples were picked for the measurements of antioxidant enzyme activities on day 90. Superoxide dismutase (SOD) activity was assayed by monitoring the inhibition of the photochemical reduction in nitroblue tetrazolium [53]. Catalase (CAT) activity was measured by monitoring the decomposition of H_2_O_2_ [54]. And peroxidase (POD) activity was determined by monitoring the oxidation of guaiacol [55].

Air-dried and sieved soil samples were used for soil enzyme activity measurements. The activity of soil urease enzyme (SUE) was determined by colorimetric determination of ammonium using assay kits from Beijing Solarbio Science & Technology Co., Ltd. (Beijing, China). Soil dehydrogenase activity (SDH) was determined with 2,3,5-triphenyltetrazolium chloride (TTC) as substrate [41]. Soil catalase (S–CAT) activity was determined by the potassium permanganate titration method [56].

The geometric mean (GMea) of detected enzyme activities was used to comprehensively characterize the enzyme activity index, which can integrate information from variables that possess different units and ranges of variation [57]:

GMea of soil enzyme = (Urease × Dehydrogenase × Catalase)^1/3^.

GMea of plant enzyme = (Superoxide dismutase × Catalase × peroxidase)^1/3^.

### 4.6. Statistical Analysis

Sample measurement and analysis were repeated at least three times in the same treatment to decrease experimental errors. The significance of differences was examined by one-way analysis of variance (ANOVA) with Duncan’s tests using SPSS 22.0. And visualization of analysis results was performed with Origin 2023. Pearson correlation analysis and the Mantel test were carried out and visualized in R (ver. 4.4.0) through the linkET, ggplot2, and dplyr packages.

## 5. Conclusions

In this study, the combined application of microbial agents and biochar was the best method to improve the phytoremediation efficiency of oil-contaminated soils by *T. erecta*. In the treatment with the co-application of plant–biochar–microbial agent, TPH degradation efficiency achieved 76.60%, and long-chain alkanes accumulated less. Analysis of plant physiological parameters revealed that microbial agents significantly reduced stress degree (plant antioxidant enzyme activities and NPQ_Lss) and improved plant growth (biomass and root activity). In addition, microbial agents also enhanced soil oxidoreductase activities better than biochar. These results demonstrated that one of the mechanisms by which microbial agents improve phytoremediation was to reduce the oil concentration in the soil through their oil degradation capacity and the increase in the soil oxidoreductase activities, so as to alleviate the stress of contamination on plants, thus promoting plant growth and phytoremediation. Whereas biochar showed insignificant correlations with most indicators and was more likely to reduce soil oil concentrations through its own porous structure and adsorption properties, the interaction effect analysis indicated a synergistic effect between microbial agents and biochar. In summary, our study concluded that the co-application of microbial agents and biochar is a promising approach to improve the phytoremediation of oil-contaminated soil and revealed possible improvement mechanisms based on the changes in the concentrations of various components of oil in the soil, the response of plants and soil enzymes, and the synergistic effect between microbial agents and biochar.

## Data Availability

Data are contained within the article.

## Supplementary Materials

The following supporting information can be downloaded at: https://www.mdpi.com/article/10.3390/plants14020243/s1, Figure S1. Chromatograms of n-alkanes extracted from soil of different treatment: (A), CK; (B), T1; (C), T2; (D), T3; (E), T4. Figure S2. Growth status of plants of different treatment: T1, T2, T3, T4.
