# Supplementary material for: Phytoremediation of Oil-Contaminated Soil by Tagetes erecta L. Combined with Biochar and Microbial Agent
Source: Plants (Basel). 2025 Jan 16;14(2):243. doi: 10.3390/plants14020243 (PMC11768401; doi:10.3390/plants14020243)
Supplement: Supplementary file 1 [file plants-14-00243-s001.zip › plants-3405455-supplementary.pdf]

Supplementary File

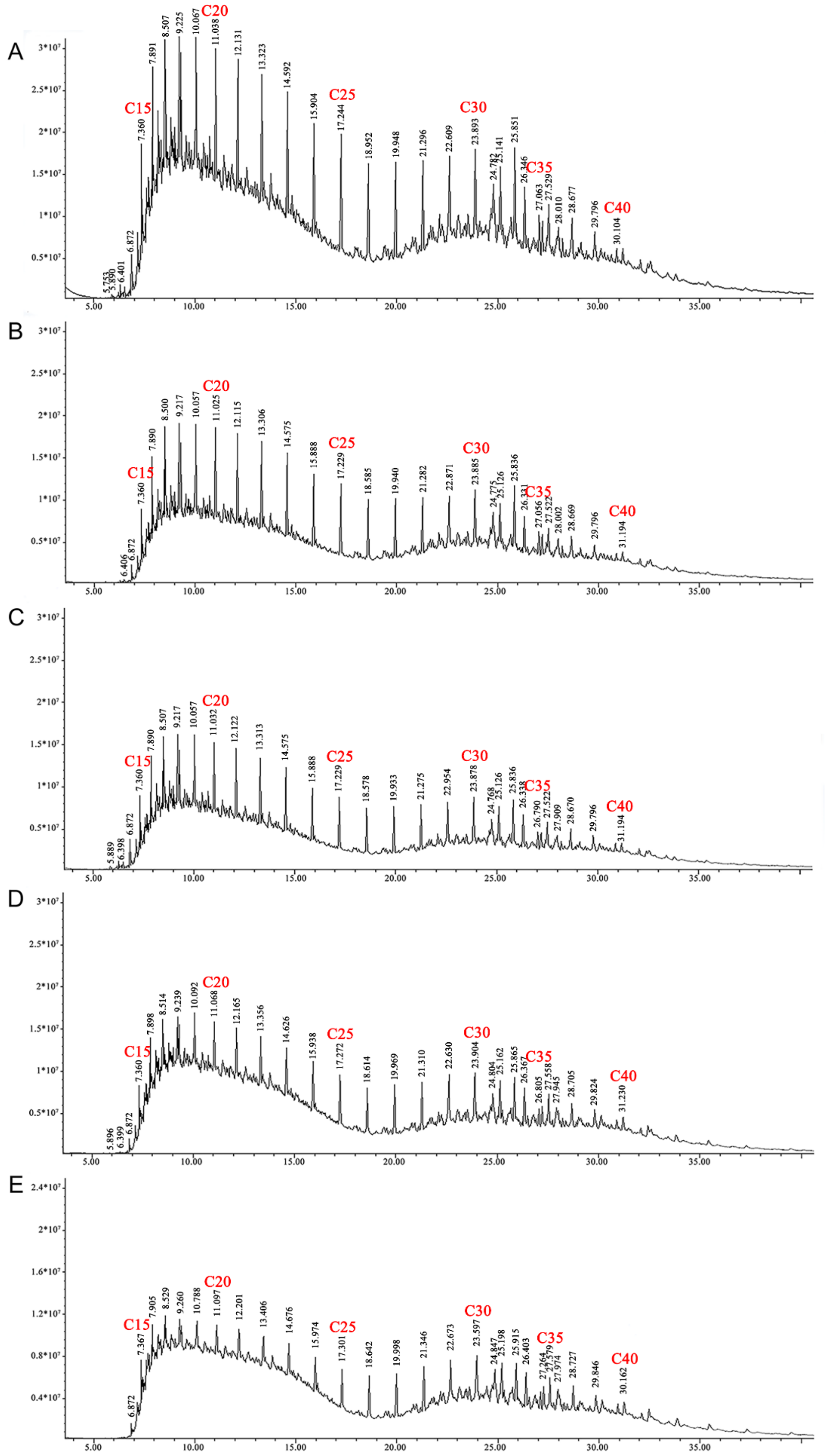

Supplementary Figure S1. Chromatograms of n–alkanes extracted from soil of different treatment: (A), CK; (B), T1; (C), T2; (D), T3; (E), T4.

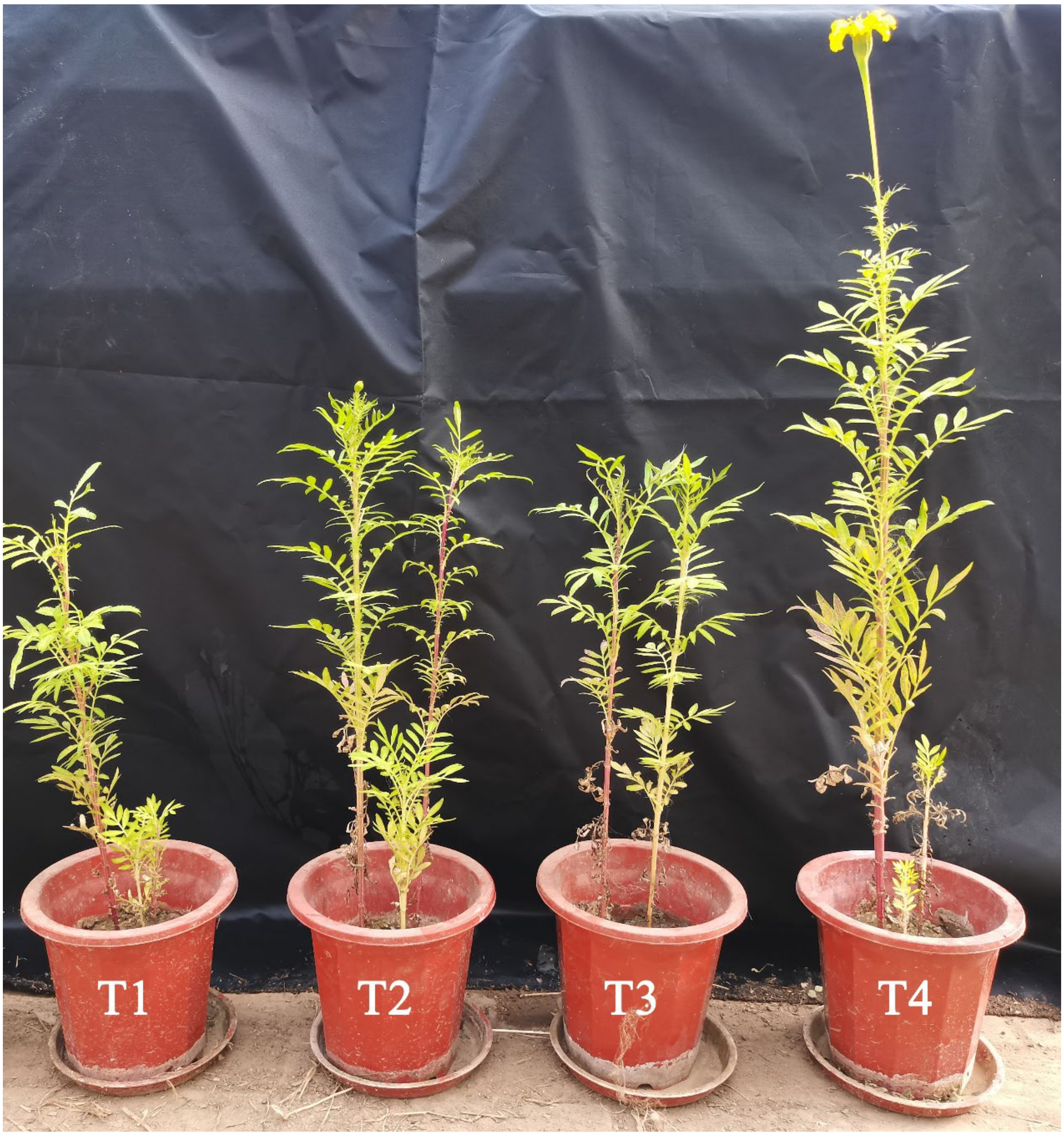

Supplementary Figure S2. Growth status of plants of different treatment: T1, T2, T3, T4.
